# Network model of skeletal muscle cell signalling predicts differential responses to endurance and resistance exercise training

**DOI:** 10.1113/EP091712

**Published:** 2024-04-21

**Authors:** Annabelle Fowler, Katherine R. Knaus, Stephanie Khuu, Ali Khalilimeybodi, Simon Schenk, Samuel R. Ward, Andrew C. Fry, Padmini Rangamani, Andrew D. McCulloch

**Affiliations:** ^1^ Department of Bioengineering University of California San Diego La Jolla California USA; ^2^ Department of Mechanical and Aerospace Engineering University of California San Diego La Jolla California USA; ^3^ Department of Orthopaedic Surgery University of California San Diego La Jolla California USA; ^4^ Department of Medicine University of California San Diego La Jolla California USA; ^5^ Department of Health, Sport and Exercise Sciences University of Kansas Lawrence Kansas USA

**Keywords:** computational model, endurance exercise, exercise, resistance exercise, signalling network, skeletal muscle

## Abstract

Exercise‐induced muscle adaptations vary based on exercise modality and intensity. We constructed a signalling network model from 87 published studies of human or rodent skeletal muscle cell responses to endurance or resistance exercise in vivo or simulated exercise in vitro. The network comprises 259 signalling interactions between 120 nodes, representing eight membrane receptors and eight canonical signalling pathways regulating 14 transcriptional regulators, 28 target genes and 12 exercise‐induced phenotypes. Using this network, we formulated a logic‐based ordinary differential equation model predicting time‐dependent molecular and phenotypic alterations following acute endurance and resistance exercises. Compared with nine independent studies, the model accurately predicted 18/21 (85%) acute responses to resistance exercise and 12/16 (75%) acute responses to endurance exercise. Detailed sensitivity analysis of differential phenotypic responses to resistance and endurance training showed that, in the model, exercise regulates cell growth and protein synthesis primarily by signalling via mechanistic target of rapamycin, which is activated by Akt and inhibited in endurance exercise by AMP‐activated protein kinase. Endurance exercise preferentially activates inflammation via reactive oxygen species and nuclear factor κB signalling. Furthermore, the expected preferential activation of mitochondrial biogenesis by endurance exercise was counterbalanced in the model by protein kinase C in response to resistance training. This model provides a new tool for investigating cross‐talk between skeletal muscle signalling pathways activated by endurance and resistance exercise, and the mechanisms of interactions such as the interference effects of endurance training on resistance exercise outcomes.

## Abstract (highlights)

- **What is the central question of this study?**
  How do the cell signalling pathways regulating skeletal myocyte responses to resistance and endurance exercise interact?
- **What is the main finding and its importance?**
  A new systems model of skeletal muscle signalling pathways activated by resistance and endurance training was developed with eight canonical signalling pathways regulating 14 transcriptional regulators, 28 target genes and 12 exercise‐induced phenotypes. The model accurately predicted 85% of independent measurements for resistance exercise and 75% for endurance training. Analysis revealed pathways regulating the preferential activation of protein synthesis and cell growth by resistance training and inflammation by endurance exercise.

## 1 INTRODUCTION

Exercise stimulates phenotypic changes in skeletal muscle, including metabolic adaptations, hypertrophy and tissue restructuring. Distinct training protocols, such as resistance, endurance and sprint exercises, activate different cell signalling pathways that lead to diverse phenotypic responses (Baar, 2006). Conventionally, resistance exercise preferentially promotes skeletal myocyte protein synthesis, culminating in muscle hypertrophy with sustained training (Qi et al., 2013). Conversely, endurance exercise primarily promotes mitochondrial biogenesis, while suppressing protein synthesis and cell growth (Qi et al., 2013). Importantly, these exercise‐induced responses are interrelated, with evidence suggesting that combining resistance and endurance training can either amplify or interfere with the effects elicited by a single exercise modality (Baar, 2006; Qi et al., 2013). Since most physical training regimens involve a combination of resistance and endurance exercises, deciphering the mechanisms driving these adaptations would enhance our ability to predict skeletal muscle phenotypic changes in response to various exercise training programmes.

Skeletal muscle responses to exercise are regulated in part by activation of signalling pathways that control gene and protein expression. Although the activation of individual pathways during exercise has been explored, the interplay between pathways that coordinate responses to varied exercise modalities remains unclear. Many of the same pathways regulate adaptations to both resistance and endurance exercise, but distinct combinations and sequences of exercise training can manifest divergent phenotypes. For example, endurance and resistance training both activate insulin signalling (Consitt et al., 2019), whereas endurance exercise preferentially activates the β‐adrenergic pathway (Sato et al., 2011). The interconnections in the exercise signalling network make it difficult to intuitively understand mechanisms of interference or synergy between different exercise modes.

Systems biology models of cell biochemical networks have previously been used to investigate pathway interactions, characterize the sensitivity of cell responses to molecular perturbations, and simulate novel experiments (Akberdin et al., 2021; Coccimiglio & Clarke, 2020; Tan et al., 2017). Here we constructed a new computational model to investigate system‐level regulation of skeletal muscle cell responses to acute resistance and endurance training. This model integrates findings from a wide range of exercise signalling studies, offering mechanistic insights into observed responses to various exercise protocols. Model simulations were used to investigate pathway interactions that mediate responses to combination training. Model outputs were corroborated with independent data not used to formulate it.

## 2 METHODS

### 2.1 Network construction

We formulated the signalling network from empirical observations reported in 87 publications, encompassing signalling activity, gene expression, and phenotypic alterations during exercise or simulated exercise in human or rodent skeletal muscle, in vivo or in vitro. Human in vivo studies informing the model formulation reported responses to resistance (squat, leg press, etc.) and endurance (e.g. cycling, treadmill running or rowing) exercises. Rodent exercise models included synergist ablation, weightlifting, and treadmill running. In vitro studies measured responses to cell stretching, or electrical or chemical stimulation to produce isometric or concentric myocyte contractions. Table 1 categorizes the references used for network construction by primary pathway and experimental system. For the purposes of building the model, we neither specifically included nor excluded data based on important variables such as age, sex or exercise duration and intensity. The selection criteria for the 87 papers used to formulate the model are summarized in a PRISMA diagram (Supporting information, Figure S1), and details of the species, muscle, exercise protocol and measurements for each of the 87 papers used to formulate the model are included in the data supplement (Supporting information, Table S2).

**TABLE 1:** References used for network reconstruction by experimental system and pathway.

System, reference type, or measurement	PI3K–Akt–mTOR	STARS	MAPK	HSP70	Hippo	NFκB	TGFβ–BMP–Smad	cAMP–AMPK	Calcium
Human resistance exercise		Lamon et al. (2009)	Silvennoinen et al. (2015)	Liu et al. (2004)		Bickel et al. (2005); Vella et al. (2012)		Silvennoinen et al. (2015)	Silvennoinen et al. (2015)
Human endurance exercise		Reitzner et al. (2018); Wallace et al. (2011)	Silvennoinen et al. (2015)	Liu et al. (2004); Morton et al. (2006)				Silvennoinen et al. (2015)	Silvennoinen et al. (2015)
Rodent synergist ablation	Martin et al. (2014); Miyazaki et al. (2011); White et al. (2014)		Carlson et al. (2001); Martin et al. (2014); Miyazaki et al. (2011)		Goodman et al. (2015)				
Rodent weightlifting	Hernandez et al. (2000)								
Rodent treadmill	Arias et al. (2001); White et al. (2014); Williamson et al. (2006)		Williamson et al. (2006)	Ogata et al. (2009)				Williamson et al. (2006)	Wu et al. (2001)
Rodent immobilization/unloading	Klossner et al. (2009)	Kim et al. (2014)		Senf et al. (2008)		Senf et al. (2008); van Gammeren et al. (2009)	Winbanks et al. (2013)		
Cell stretch/stimulation	Jacobs et al. (2013); Liu et al. (2013); Sherwood et al. (1999)	Zhang et al. (2007)	Carrasco et al. (2003); Liu et al. (2013); Sherwood et al. (1999)	Jorquera et al. (2009)	Wada et al. (2011)			Carrasco et al. (2003)	Carrasco et al. (2003); Eltit et al. (2006); Jorquera et al. (2009); Wu et al. (2001)
Inhibition/over‐expression	Murga et al. (2000, 1998); Haddad and Adams (2004); Roux et al. (2007); Mizutani et al. (2009)	Arai et al. (2002); Charvet et al. (2006); Kumar et al. (2006); Sotiropoulos et al. (1999); Schratt et al. (2002); Zhang et al. (2007)	Bouzakri and Zierath (2007); Cho and Gruol (2008); Janknecht et al. (1993); Li et al. (2005); Long et al. (2011); Roux et al. (2007)	Senf et al. (2008)	Han et al. (2018); Kim et al. (2013); Yu et al. (2013, 2012); Zhao et al. (2007)	Cai et al. (2004); van Gammeren et al. (2009); Tullai et al. (2011)	Engel et al. (1999); Winbanks et al. (2013); Zhang et al. (1998)	Kim et al. (2013)	Cho and Gruol (2008); Macián et al. (2000); Minetti et al. (2011)
Protein activity/modification	Miyazaki et al. (2011); Williamson et al. (2006); Klossner et al. (2009); Jacobs et al. (2013); Roux et al. (2007); Coolican et al. (1997); Liu et al. (2013); Silvennoinen et al. (2015); White et al. (2014); Murga et al. (2000); Murga et al. (1998); Mizutani et al. (2009)	Zhang et al. (2007); Kim et al. (2014); Kumar et al. (2006)	Martin et al. (2014); Miyazaki et al. (2011); Liu et al. (2013); Carlson et al. (2001); Bouzakri and Zierath (2007); Janknecht et al. (1993)		Goodman et al. (2015); Wada et al. (2011); Yu et al. (2012); Zhao et al. (2007); Han et al. (2018); Kim et al. (2014); Yu et al. (2013)	Van Gammeren et al. (2009); Cai et al. (2004); Vella et al. (2012)	Winbanks et al. (2013)	Williamson et al. (2006); Kim et al. (2014)	Koulmann and Bigard (2006); Arias et al. (2001); Winbanks et al. (2013); Zhang et al. (1998)
Total protein	Klossner et al. (2009); Long et al. (2011); White et al. (2014); Arias et al. (2001)	Zhang et al. (2007); Charvet et al. (2006); Lamon et al. (2009); Schratt et al. (2002); Reitzner et al. (2018); Wallace et al. (2011)	Cho and Gruol (2008)	Liu et al. (1999); Senf et al. (2008); Jorquera et al. (2009); Morton et al. (2006); Ogata et al. (2009)	Goodman et al. (2015)				Jorquera et al. (2009); Cho and Gruol (2008)
mRNA expression	Long et al. (2011); Silvennoinen et al. (2015); White et al. (2014)	Charvet et al. (2006); Lamon et al. (2009); Schratt et al. (2002); Reitzner et al. (2018); Wallace et al. (2011)	Liu et al. (2013); Janknecht et al. (1993)	Figueir et al. (2015); Cai et al. (2004); Jorquera et al. (2009); Hernandez et al. (2000)	Goodman et al. (2015)	Lamon et al. (2014); Yu et al. (2013)			Carrasco et al. (2003); Jorquera et al. (2009); Silvennoinen et al. (2015); Minetti et al. (2011)
Review	Mayr and Montminy (2001); Graham et al. (2015); McGlory et al. (2017)	Miano et al. (2007); Lamon et al. (2014); Graham et al. (2015)	Whitmarsh and Davis (1996); Kramer and Goodyear (2007); He et al. (2016)		Halder et al. (2012); Meng et al. (2016); Fischer et al. (2016); Gabriel et al. (2016); Watt et al. (2018)	Febbraio and Pedersen (2002); Kramer and Goodyear (2007); Bakkar and Guttridge (2010); Xu et al. (2017)	Elkina et al. (2011); Goodman and Hornberger (2014); Gumucio et al. (2015); Borok et al. (2020)	Mayr and Montminy (2001); Wen et al. (2010); Hardie (2011)	Koulmann and Bigard (2006); Berdeaux and Stewart (2012); Kang and Li Ji (2012); Benavides Damm and Egli (2014); Graham et al. (2015); McGlory et al. (2017); Mirzoev et al. (2021)

To construct the network, we began by selecting studies that identified key signalling molecules involved in regulating phenotypic adaptations to exercise in skeletal muscle. Interactions between signalling nodes were based on exercise or non‐exercise studies in skeletal muscle. Model outputs included consensus gene and protein markers of exercise response or well recognized exercise‐induced phenotypic alterations in skeletal muscle.

Five of the studies used to formulate the model used the rodent synergist ablation model (Carlson et al., 2001; Goodman et al., 2015; Martin et al., 2014; Miyazaki et al., 2011; White et al., 2014), which stimulates repair responses that are not necessarily induced by physiological exercise training. The only reaction in the model that relies solely on data from this model is the activation of S6 kinase by c‐Jun N‐terminal kinase (JNK) (Martin et al., 2014). This study was one of over a dozen used to formulate the mitogen‐activated protein kinase (MAPK) pathway in the network model.

### 2.2 Logic‐based ordinary differential equation model formulation

Reactions between nodes were modelled using logic‐based ordinary differential equations (ODEs), a method previously used to model other myocyte signalling networks (Tan et al., 2017). A system of ODEs is generated from the reaction network and solved to compute the activity of each node for prescribed initial conditions and input exercise time courses. The activity of each node is governed by an ODE and varies between 0 and 1 following a saturating Hill‐type function (Tan et al., 2017). Regulation by more than one upstream node is represented using continuous versions of logical operations, where OR reactions mean activation of either upstream node is sufficient to activate a response whereas AND reactions require both to be activated.

As assumed in previous analyses (Tan et al., 2017), the same default network parameters were used for all reactions: Hill coefficient *n*_H_ = 1.4, half‐maximal activation EC_50_ = 0.5, initial activity *Y*_init_ = 0, and maximal activity *Y*_max_ = 1. The weight of all reactions was set 0.7 to limit saturation. Time constants τ in the model were chosen to be 0.1 min for receptor activation, 10 min for all signalling reactions, and 60 min for all mRNA expression reactions.

**FIGURE 1:**
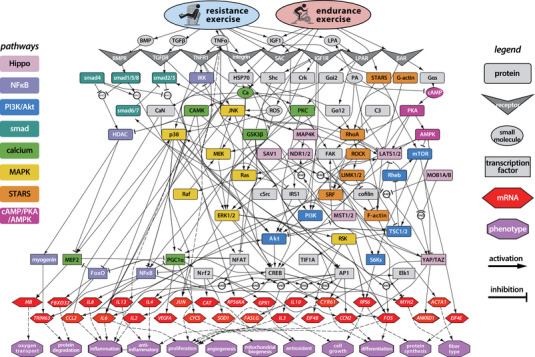
Logic‐based network model of skeletal myocyte signalling responses to resistance and endurance exercise. Schematic illustration showing 259 interactions between 120 nodes that regulate the expression of 28 genes (red hexagons) and 12 exercise‐related phenotypic outputs (purple octagons). The model includes five extracellular ligands, eight cell surface receptors, eight canonical signalling pathways, and 14 transcription factors (rectangles with hard corners). Note that multiple activating node stimuli are treated with OR logic except where AND interactions are shown.

For the simulations described herein, initial conditions were obtained by running the model with no exercise input for a simulation time of 15 h, when node activities had reached steady state. Resistance or endurance exercise was simulated by adjusting the input values *Y*_max_ for the two exercise nodes between 0 and 1. The exercise input nodes each activate ligands, receptors or signalling molecules in the network, as shown in Figure 1. The Python code and parameter sets used to generate the solutions reported here are included in a Jupyter notebook (Supporting information, Supplement S3, available in a public repository: https://doi.org/10.5281/zenodo.10257879).

### 2.3 Model validation

Model predictions were validated by comparing outputs with independent experimental results from papers not used to build the network model. In total, 37 results from nine papers were used to validate the model predictions. Validation study selection criteria are summarized in Supporting information Figure S1. Eight studies measured responses to resistance exercise and six reported responses to endurance exercise (Aronson et al., 1997; Camera et al., 2010; Figueir et al., 2015; Galpin et al., 2012; Lessard et al., 2018; Liu et al., 1999; Louis et al., 2007; Vissing, McGee et al., 2013; Vissing, Rahbek et al., 2013). All of the studies used for validation satisfied the same selection criteria as the model formulation studies with the additional requirements that they reported measurements of one of protein phosphorylation, total protein or gene expression from biopsies after a single bout of resistance or endurance exercise of similar duration in human subjects. Endurance exercise bouts ranged from 120 min at 60% to 30 min at 75% of peak or maximum ${\dot{V}}_{O_{2}}$, or to exhaustion. Resistance exercise sessions ranged from 6 to 60 contractions at 70%–100% of single repetition maximum load. To simplify comparison, we standardized the model resistance or endurance exercise input stimuli to 45 min at 1.0 (100%), while recognizing that humans cannot sustain 100% exercise output for this long. The studies used for model validation were chosen so that key nodes from all the pathways in the model could be tested. The validation studies included data from 140 human subjects of both sexes. All subjects were described as young and healthy, but only seven were female. Twenty‐one resistance exercise measurements and 16 endurance exercise measurements were used. Details of the muscle, exercise protocol, and measurements for each of the nine papers used for model validation are also included in the data supplement (Supporting information, Table S2).

We simulated 45 min of maximum resistance or endurance exercise input, using the steady‐state baseline values as the initial conditions. We then compared activity of key proteins immediately following exercise to their baseline values. Differences between exercise and baseline activity were classified as increased, decreased, or no change, using a relative change threshold in the model of 0.05. These predictions were then compared with statistically significant experimental findings from the validation papers to assess model accuracy.

### 2.4 Model sensitivity to single exercise modes

We performed sensitivity analysis to identify major nodes responsible for regulating gene expression. We simulated knockdown of each node in the network by reducing *Y*_max_ by 50% (Ryall et al., 2012; Tan et al., 2017) and predicted changes in activity of all other nodes for both resistance and endurance exercise conditions. To do this, we simulated 30 min of exercise with each node knocked down and subtracted activation values from model predictions of 30 min of exercise with no nodes knocked down. Nodes in the network causing the greatest total changes in activity were identified as key regulators of exercise response.

### 2.5 Combining exercise modes

We simulated 45 min of resistance exercise (input stimulus = 1), followed immediately by 45 min of endurance exercise (input stimulus = 1), as well as the reverse order. Additionally, we simulated 90 min each of resistance and endurance exercise alone starting from a baseline level of zero. We compared relative changes in phenotypic activity after exercise to outputs after 90 min of model simulation with a constant baseline (0.1) exercise input, to determine differences in responses to resistance, endurance and concurrent training.

We then repeated both concurrent exercise simulations, with AMP‐activated protein kinase (AMPK) knocked down by reducing *Y*_max_ to 0.1, and again with simulated tumour necrosis factor α (TNFα) and reactive oxygen species (ROS) knockdown.

## 3 RESULTS

### 3.1 Predictive computational model of exercise signalling network in skeletal muscle

The model network has 120 nodes and 259 reactions (Figure 1 and Supporting information Table S4) representing eight key pathways that regulate skeletal myocyte responses to exercise, as well as the crosstalk between them. Model outputs include the expression of 28 genes commonly measured, all of which have been identified as markers of exercise‐related skeletal muscle phenotypes. The model also has 12 generic, transcriptionally regulated phenotypic outputs: protein synthesis and degradation, proliferation, differentiation, cell growth, mitochondrial biogenesis, angiogenesis, oxygen transport, inflammation and anti‐inflammatory, antioxidant production, and changes in fibre type.

Several of the model pathways regulate skeletal muscle hypertrophic responses. Resistance exercise in the model activates the transforming growth factor β (TGF‐β) and bone morphogenic protein (BMP) receptors engaging the Smad signalling pathway (shown in teal in Figure 1) that regulates protein synthesis and myocyte growth by inhibiting Akt (Borok et al., 2020; Goodman & Hornberger, 2014; Gumucio et al., 2015). Smads 1 and 7 and Akt also interact with Yes‐associated protein (YAP) and transcriptional coactivator with PDZ‐binding motif (TAZ) (Figure 1, pink), which regulate cell migration, growth, differentiation and proliferation (Bakkar & Guttridge, 2010; Fischer et al., 2016; Goodman et al., 2015; Halder et al., 2012; Han et al., 2018; Meng et al., 2016; Wada et al., 2011; Watt et al., 2018; Yu et al., 2012; Zhao et al., 2007).

The phosphoinositide 3‐kinase (PI3K)–Akt–mechanistic target of rapamycin (mTOR) pathway (Figure 1, blue) regulates cell growth and protein synthesis rates via p70 ribosomal S6 kinase 1 and eukaryotic initiation factor 4E binding protein‐1 (eIF4E) (Figueir et al., 2015; Jacobs et al., 2013; Martin et al., 2014; Miyazaki et al., 2011; Williamson et al., 2006). It is primarily activated in the model by insulin‐like growth factor (IGF1) and calcium (Ca) in response to resistance and endurance exercise (Benavides Damm & Egli, 2014; Coolican et al., 1997; Florini et al., 1996; Jacobs et al., 2013; Klossner et al., 2009; Martin et al., 2014; Miyazaki et al., 2011; Roux et al., 2007; Williamson et al., 2006; Zhang et al., 2007).

The MAPK pathway (Figure 1, yellow) can also activate rpS6 independently of mTOR, and ribosomal S6 kinase (RSK) phosphorylates S6 directly (Figueir et al., 2015; Liu et al., 2013; Martin et al., 2014; Roux et al., 2007; Williamson et al., 2006). RSK, extracellular signal‐regulated kinase (ERK), JNK, and p38 regulate transcription factors including cAMP response element‐binding protein (CREB), peroxisome proliferator‐activated receptor γ coactivator 1‐α (PGC1α), and ETS‐like gene 1 (Elk1) and downstream phenotypes including cell proliferation and differentiation (Carrasco et al., 2003; Figueir et al., 2015; Long et al., 2011).

The striated muscle activator of Rho signalling (STARS) pathway (Figure 1, orange) regulates the transcriptional activity of serum response factor (SRF) via actin dynamics and RhoA signalling. STARS is responsive to both endurance and resistance exercise. SRF regulates genes in the model associated with skeletal muscle cell differentiation, proliferation and growth (Arai et al., 2002; Charvet et al., 2006; Kim et al., 2014; Lamon et al., 2009, 2014; Miano et al., 2007; Schratt et al., 2002; Sotiropoulos et al., 1999; Vissing, Rahbek et al., 2013; Zhao et al., 2007).

The model also includes inflammatory responses to exercise. The expression of inflammatory myokine genes *IL6*, *IL8* and *CCL2*, as well as *TRIM63*, which leads to protein degradation, is transcriptionally regulated by nuclear factor κB (NFκB; Figure 1, purple), which is activated in the model by the IkappaB kinase (IKK) complex, in response to TNFα receptor stimulation during resistance exercise (Bakkar & Guttridge, 2010; Cai et al., 2004; van Gammeren et al., 2009; Vella et al., 2012).

Heat shock protein‐70 (HSP70/HSP72) is activated in the model both by resistance and endurance exercise and acts to inhibit forkhead box O (FoxO) and NF𝜅B activity.(Senf et al., 2008). It is expressed in skeletal muscle in response to exercise‐related stresses such as increased temperature, glycogen depletion, pH changes, calcium signalling and increased levels of reactive oxygen species (Jorquera et al., 2009; Liu et al., 2004, 1999).

Finally, several pathways in the model regulate metabolic activity. Calcium signalling (Figure 1, green) (Koulmann & Bigard, 2006) activates calcineurin (CaN), protein kinase C (PKC), and calcium calmodulin kinase (CAMK), which control PGC1𝛼 and myocyte enhancer factor‐2 (MEF2), regulating gene expression that affects oxygen transport, fibre type, mitochondrial biogenesis and angiogenesis.

Endurance exercise activates the cAMP–protein kinase A (PKA)–AMPK pathway (Figure 1, magenta) via the β‐adrenergic receptor (βAR). AMPK inhibits mTOR (Williamson et al., 2006), decreasing global rates of muscle protein synthesis and cell growth, and activates PGC1𝛼, increasing mitochondrial biogenesis (Hardie, 2011).

**FIGURE 2:**
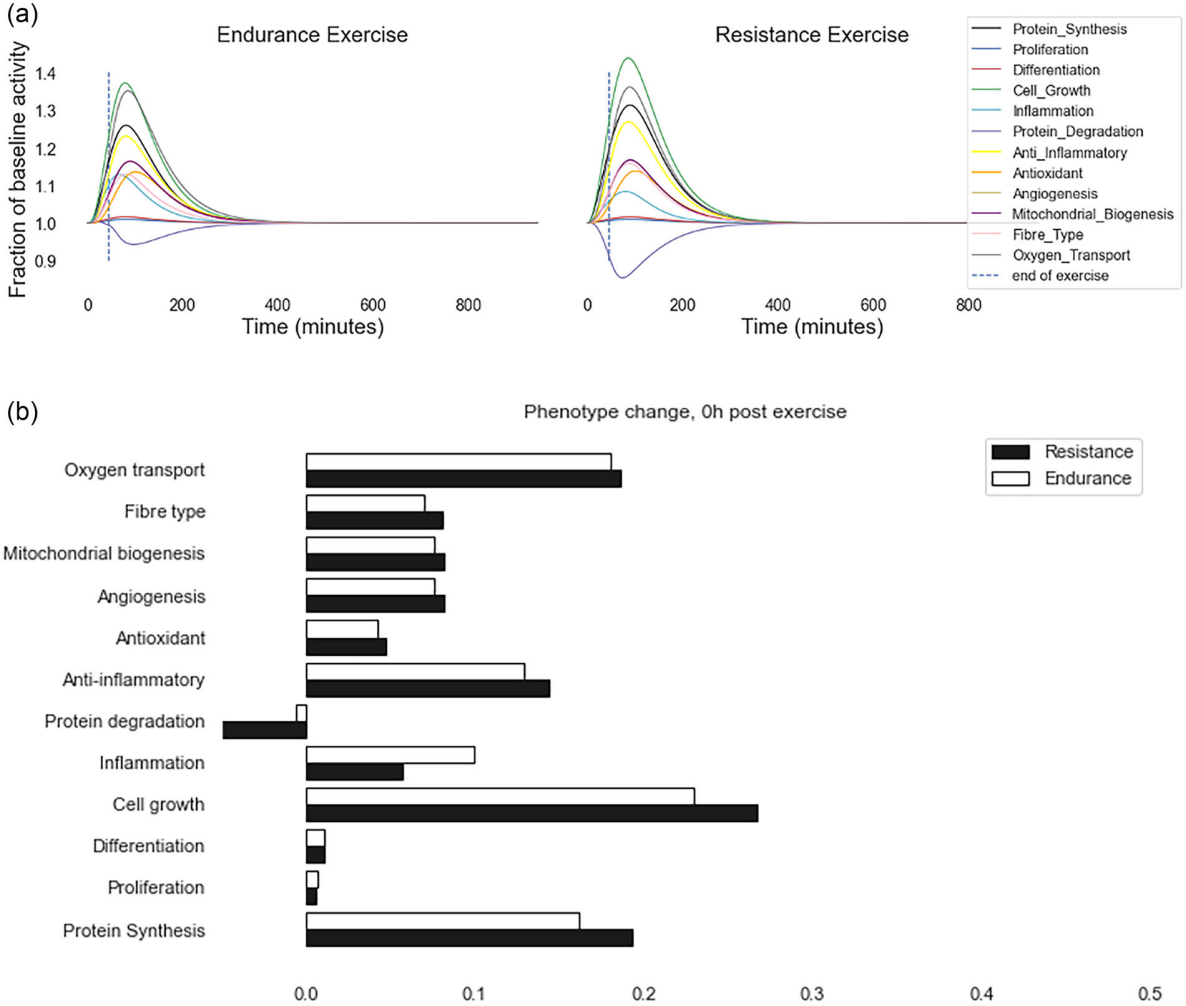
Model‐predicted changes in phenotypes relative to baseline during 45 min of exercise and 15 h recovery. (a) Time courses of phenotypes as a fraction of baseline for endurance (left) and resistance (right) exercise. Vertical dashed line represents end of exercise bout. (b) Fractional changes from 1.0 (baseline) in each phenotype immediately after exercise showing greatest differences between responses to resistance and endurance training in protein degradation, inflammation, cell growth and protein synthesis.

To determine the effect of acute exercise on determinants of phenotypic change, we ran the model to steady state with no exercise input, followed by a simulated bout of maximal resistance or endurance exercise (input = 1.0) for 45 min and 15 h of rest post‐exercise. The expression of most genes in the model increased during exercise, returning toward baseline following cessation (Figure 2a,b), though a significant number of genes remained significantly upregulated after 15 h of rest. The model predicted differences between resistance and endurance exercise in the acute expression of genes associated with inflammation, protein turnover and cell growth phenotypes. By 15 h the differences in muscle phenotypes between exercise modality were negligible. Differences between 15 and 24 h were also generally low.

### 3.2 Model validation for different exercise modes

**FIGURE 3:**
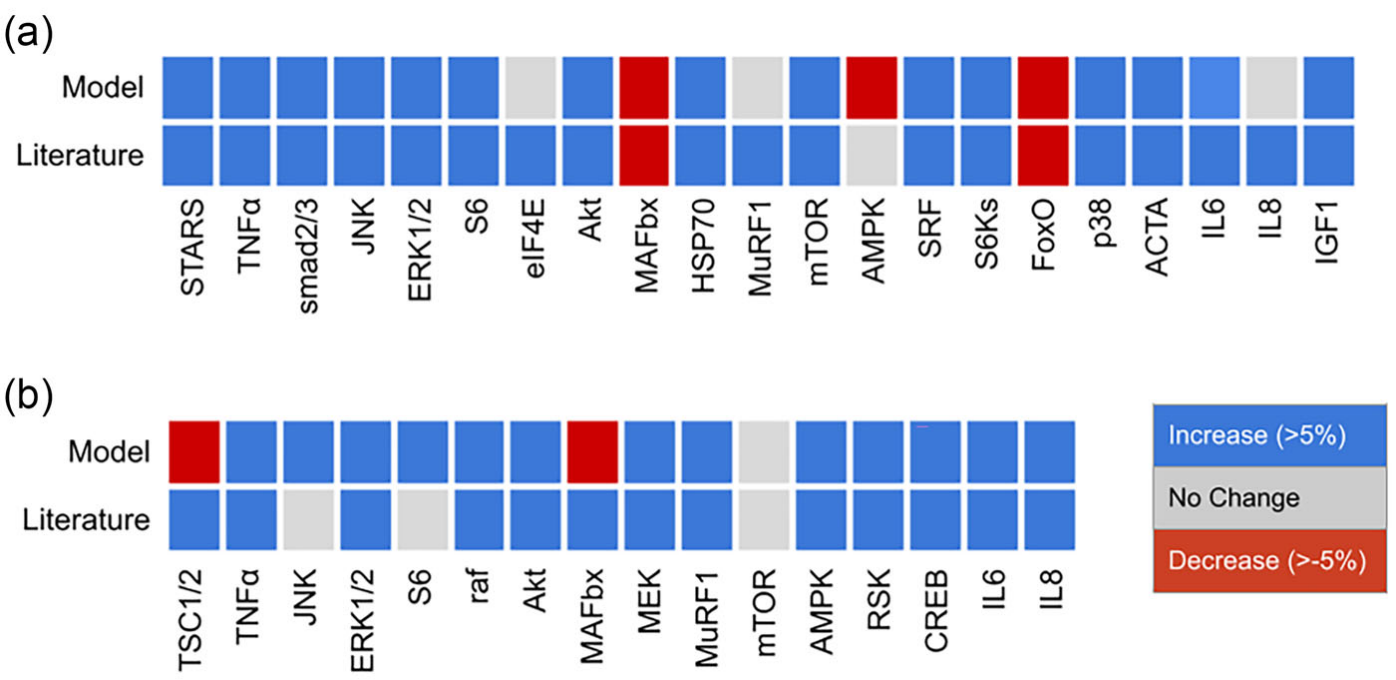
Model validation. Simulated (Model) fractional changes from baseline in network node activity after 45 min of exercise (input = 1) compared with validation results (literature) from published experimental measurements in muscle that were not used to formulate the model. Validation results are shown in red for a reported statistically significant decrease during exercise, blue for a reported statistically significant increase, and grey for no significant change. Model nodes were deemed to be increased when the node variable increased from baseline by more than 0.05, decreased when the variable decreased by more than 0.05 from baseline, or unchanged otherwise. The model correctly predicts 18 of 21 responses to resistance exercise (a) and 12 of 16 responses to endurance exercise (b).

Compared with experimental results from papers not used to construct the signalling network, the model accurately predicted 85% (18/21) measurements of resistance exercise responses and 75% (12/16) measurements of endurance exercise responses (Figure 3). Three of the seven discrepancies were instances where the model variable changed by more than the threshold (5% of baseline) but measurements reported no significant changes. In another three comparisons, the model predicted decreases (in muscle RING‐finger protein‐1 (MuRF1) in response to resistance exercise, and in TSC1/2 and muscle atrophy F‐box (MAFbx) in response to endurance exercise), whereas experimental studies reported a significant increase. To assess the sensitivity of model accuracy to parameter uncertainty, we repeated the validation analysis by perturbing the reaction parameters. When *n*_H_ and EC_50_ were increased from 1.4 and 0.5 to 2.0 and 0.6, respectively, validation accuracy for model predictions decreased from 81% to 76% for resistance exercise and from 75% to 69% for endurance exercise. When *n*_H_ and EC_50_ were decreased to 1.0 and 0.4, respectively, model accuracy decreased to 62% for resistance exercise and 50% for endurance exercise. Hence, the model results were quite robust.

### 3.3 Identifying key regulators in different exercise modes

Network sensitivity analysis identified the STARS, calcium, TNF𝛼, MAPK, cAMP–AMPK and PI3K–Akt–mTOR pathways as the most important regulators of response to resistance exercise (Figure 4a and Supporting information Table S5A). These were the nodes that, when knocked down, caused the greatest change in activity of phenotypes in the network (sum of absolute values of rows of the sensitivity matrix >1.5). These pathways promote protein synthesis, cell growth, inflammation and mitochondrial biogenesis.

FIGURE 4Heat maps showing sensitivity of 12 model output phenotypic responses (rows) when each network node (column) is individually knocked down by 50%. Phenotypes are ordered from most responsive to resistance exercise at top (blue) to most responsive to endurance exercise at bottom (red). Knocked down nodes are group by their category or pathway shown in Figure 1 and ordered as follows: receptors (SAC, LPA, lysophosphatidic acid (LPAR), βAR, BMP, BMPR, IGF1, IGF1R, TGFβ, TGFβR, TNF𝛼, TNFR1, integrin); calcium (Ca, CaMK, PKC, CaN, GSK3β, Shc, Crk); smad (smad2/3, smad4, smad1/5/8, smad6/7); STARS (STARS, F‐actin, G‐actin, RhoA, LIMK1/2, ROCK, SRF); Hippo (PA, MAP4K, MST1/2, SAV1, MOB1A/B, LATS1/2, NDR1/2); PI3K/Akt (PI3K, Akt, TSC1/2, mTOR, Rheb) MAP kinase (Ras, Raf, p38, JNK, MEK, ERK1/2, RSK); NF𝜅B (IKK, HDAC); cAMP/AMPK/PKA (cAMP, AMPK, PKA); Other: (HSP70, G𝛼12, G𝛼_i_2, G𝛼_s_, ROS); and transcription factors (YAP/TAZ, myogenin, S6Ks, FoxO, NF𝜅B, CREB, AP1, TIF1A, Elk1, Nrf2, MEF2, PGC1𝛼, NFAT). The heat map scale represents knock‐down response minus baseline response with blue >0 and red <0. (a) Sensitivity of phenotypes to knockdowns during resistance exercise. (b) Sensitivity of phenotypes to knockdowns during resistance exercise. (c) Output phenotype sensitivities during resistance exercise minus phenotype sensitivities during resistance exercise. For original data used for these maps, see Supporting informatiion Table S4A–C.

**Figure:**
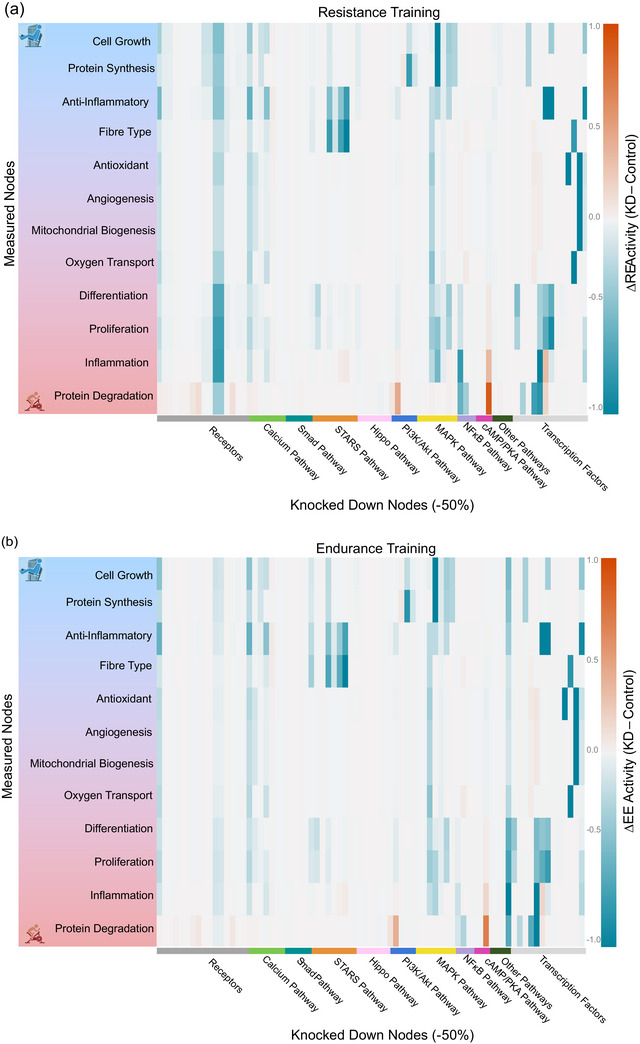

**Figure:**
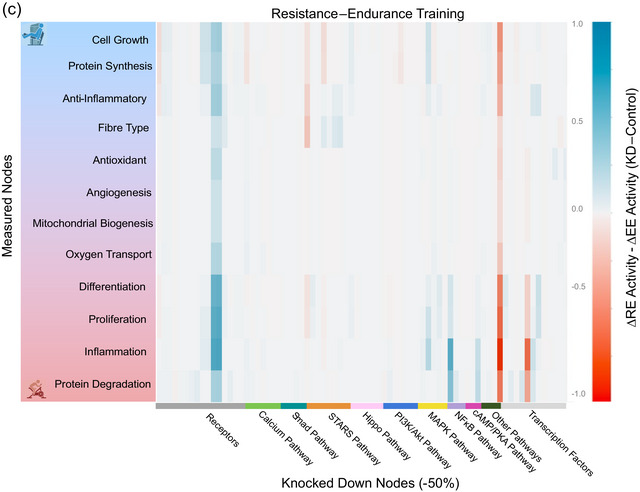

In the case of endurance exercise (Figure 4b and Supporting information Table S5B), nodes of the STARS, ROS, calcium, TNF𝛼, MAPK, cAMP–AMPK and PI3K–Akt–mTOR pathways were identified as important. Examining the differences between node sensitivities during resistance and endurance exercise (Figure 4c and Supporting information Table S5C) reveals that the most important mediators of the differences between resistance and endurance training responses were MAPK and mTOR promotion of cell growth and protein synthesis, and ROS and NF𝜅B activation of inflammation and protein degradation. Using a 100% knockout instead of a 50% knockdown of nodes in the sensitivity analysis did not change these conclusions.

### 3.4 Combining exercise modes

When we simulated concurrent training (45 min each of resistance and endurance exercise), we found that the order of training sessions did not significantly impact peak phenotypic alterations following exercise; however, the timing of the peaks was relative to the timing of the primary exercise stimulus regulating the phenotype. Concurrent training elicited increases in protein synthesis and degradation, cell growth and anti‐inflammatory activity that were greater than those induced by endurance training alone but smaller than those due to resistance training alone.

**FIGURE 5:**
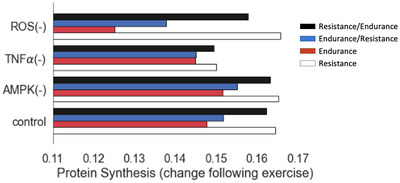
Predicted changes in protein synthesis phenotypes following resistance, endurance and concurrent training. Effects of knocking down AMPK, TNF𝛼 and ROS on differential exercise responses. AMPK knockdown had little effect on these differences, while ROS knockdown exaggerated them and TNF𝛼 knockdown largely eliminated them.

To determine whether suppression of PI3K–Akt–mTOR signalling by AMPK is responsible for diminished protein synthesis after concurrent training, we re‐ran the combined exercise simulations, with *Y*_max_ of AMPK reduced to 0. Knocking down AMPK increased protein synthesis after resistance, endurance and concurrent training; however, the magnitudes of these differences were very small, indicating that this is not the primary mechanism driving this effect in the model (Figure 5). We also simulated the knockdown of TNFα and ROS. We found that knocking down TNFα largely eliminated the differences in protein synthesis between exercise modes. In contrast, knocking down ROS increased the observed differences between resistance and endurance exercise responses compared with control. Repeating the analysis in Figure 5 with 100% knockout instead of 90% knockdown of AMPK, TNFα and ROS resulted in negligible differences.

## 4 DISCUSSION

This new model of skeletal myocyte exercise signalling provides mechanistic insight into the differential phenotypic responses to two primary modes of exercise training. The model includes 120 nodes connected by 259 reactions, and it predicts changes in 12 phenotypic outcomes in response to resistance and endurance exercise inputs. The model accurately predicted 85% of resistance and 75% of endurance exercise measurements from independent studies.

The activity of all phenotypic outputs changed in response to both exercise inputs; however, the magnitude of change differs between resistance and endurance exercise. In particular, the model predicted differences in activity of genes related to inflammation, protein synthesis, cell growth and protein degradation during acute exercise between resistance and endurance. These results suggest that the model recapitulates well‐known differences between the effects of resistance and endurance exercise training on skeletal muscle signalling (Vissing, McGee et al., 2013).

**FIGURE 6:**
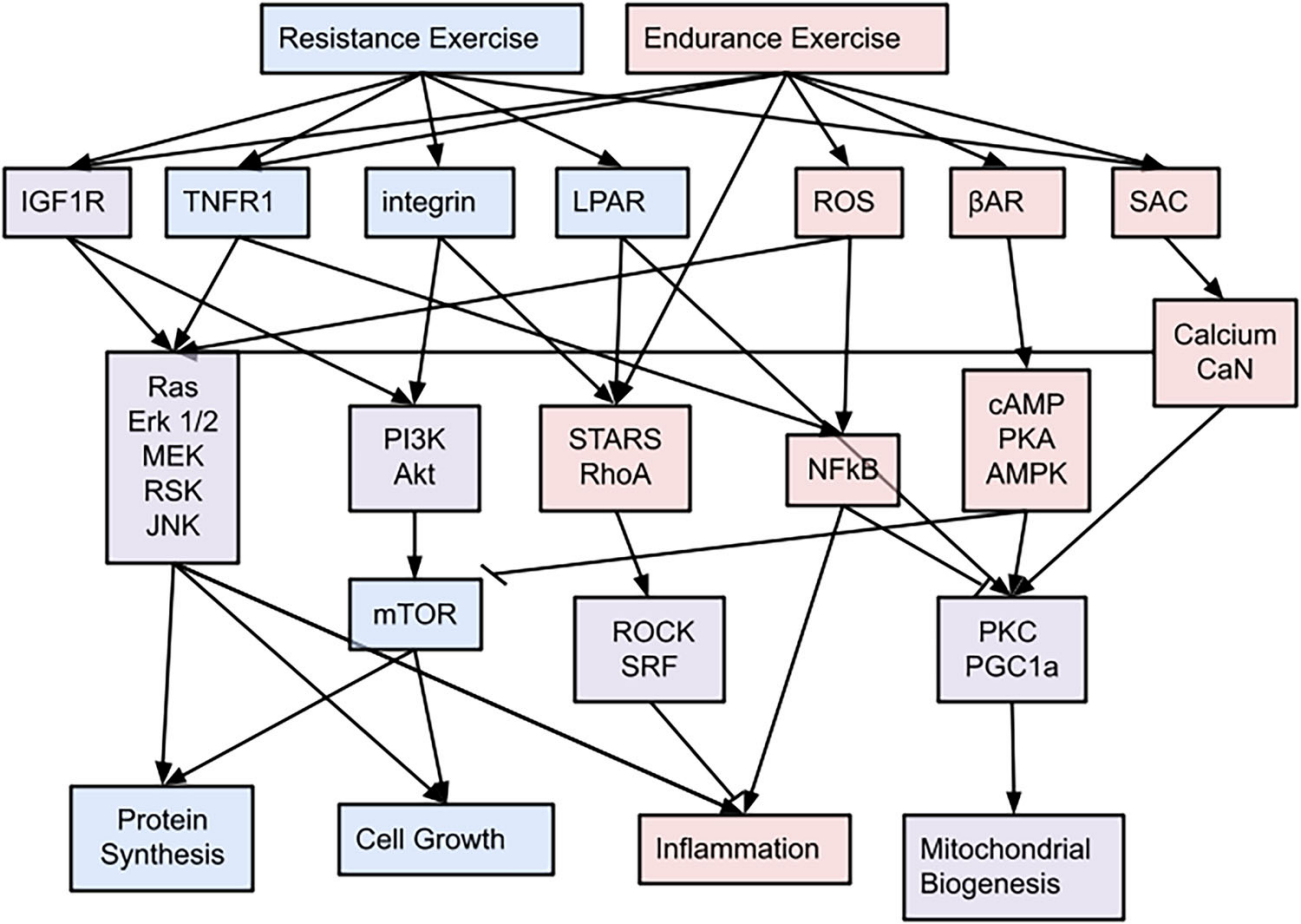
Simplified network diagram showing key nodes and pathways regulating responses to resistance and endurance exercise. Resistance exercise regulates cell growth and protein synthesis primarily by signalling via mTOR, which is activated by Akt and inhibited in endurance exercise by AMPK. Endurance exercise preferentially activates inflammation via ROS and NFkB signalling. The expected preferential activation of mitochondrial biogenesis by endurance exercise was counterbalanced in the model by LPR regulation of PKC in response to resistance training.

Sensitivity analysis identified key nodes and pathways regulating responses to resistance and endurance exercise in the model (Figure 6). We found that the MAP kinase, PI3 kinase, STARS, NF𝜅B, cyclic AMP, and calcium pathways are particularly important regulators of responses to both forms of exercise. Many of the same signalling cascades drive responses to both resistance and endurance exercise, but the magnitude of activation differs between modes. Greater predicted inflammation following endurance exercise resulted from NF𝜅B activation by ROS in endurance exercise. It is worth noting that inflammation in the studies we used to formulate the model may have been a stress or damage response in naive subjects as opposed to an adaptive response in trained subjects. Resistance exercise preferentially regulated cell growth and protein synthesis primarily via mTOR signalling activated by Akt and inhibited in endurance exercise by AMPK. Interestingly, the differences in protein synthesis between resistance, endurance and concurrent training in protein synthesis rates were largely eliminated by knocking down TNF𝛼 in the model. Inhibiting ROS reduced protein synthesis activated by endurance exercise but had no effect on protein synthesis activated by resistance exercise. While, high doses of non‐steroidal anti‐inflammatory drugs have been reported to interfere with muscle hypertrophy stimulated by resistance training, a recent study concluded that well recognized regulators of protein synthesis during resistance exercise, like those included in our model, do not explain this observation (Lilja *et al.*, 2023). Finally, the model failed to predict the expected preferential activation of mitochondrial biogenesis by endurance exercise. Although PGC1𝛼 is activated by AMPK and calcium signalling in endurance exercise, inhibition of PGC1𝛼 by NF𝜅B by endurance training and activation of PKC by LPA during resistance exercise counteracted the differences between endurance and resistance training on mitochondrial biogenesis in the model.

### 4.1 Mechanisms of differential effects of resistance, endurance and concurrent training

Most athletes employ concurrent training, that is, the combination of resistance and endurance exercise training, to improve strength, power and endurance (Baar, 2006). However, the interactions between endurance and resistance exercise signalling pathways and how they are affected by the timing, duration and intensity of exercise remain poorly understood.(Inoue et al., 2016). An interference effect has been described, wherein the muscle hypertrophy due to concurrent training is less than that resulting from resistance training alone (Mesquita et al., 2021). Hypothesized mechanisms have included repression of PI3K–Akt–mTOR signalling and ribosome biogenesis by AMPK activation during glycogen‐depleting endurance exercise (Mesquita et al., 2021).

Compared with baseline, we found increases in protein synthesis, cell growth and anti‐inflammatory activity tended to be greater with concurrent training than endurance training alone, but less than those resulting from resistance training alone. Simulating ROS knockdown decreased the effects of endurance training and concurrent training on protein synthesis while slightly increasing the effects of resistance training alone. In contrast, knocking down TNF𝛼 blunted the effect of resistance training on protein synthesis so that it was similar to the effects of endurance training. TNF𝛼 activation of MAPK signalling, S6K and rpS6 may be important in regulating differential protein synthetic responses to resistance and endurance exercise. AMPK knockdown had little effect on the differences in protein synthesis rates induced by the different exercise modes. Apró et al. (2013) reported that activation of mechanistic target of rapamycin complex 1 (mTORC1) by resistance exercise was not impaired by subsequent concurrent endurance exercise, but they also found that phosphorylation of AMPK was decreased 3 h after both resistance exercise‐only and concurrent exercise, suggesting that prior activation of mTORC may suppress AMPK activation. Our findings are consistent with those of Jørgensen and co‐workers (Jørgensen et al., 2005), who found that knocking out the 𝛼_2_ isoform but not the 𝛼_1_ isoform of AMPK decreased AMPK activation due to running, but that neither knockout affected running induced changes in gene expression.

For the short exercise protocols tested in this study, we did not see significant differences in peak magnitude of changes associated with the order of concurrent training, as have been described in some studies (Coffey & Hawley, 2017). This may be because the model does not account for the metabolic costs of exercise and the fact that mitochondrial biogenesis and protein turnover require more cellular energy than is needed for metabolic homeostasis. It would be useful to couple this model with a model of skeletal myocyte energy metabolism, which is well established (Dash et al., 2007). One recent report describes a model of skeletal muscle that combines energy metabolism, calcium and AMPK signalling pathways with gene expression (Akberdin et al., 2021).

These findings highlight the potential of the model for screening a variety of different exercise protocols for differential phenotypic responses, to generate new hypotheses that can then be tested in vivo.

### 4.2 Limitations

In response to resistance exercise, the model predicted a decrease in AMPK, which was not reported in the published validation studies. The model also failed to predict an increase in *IL8 and eiF4E* expression in response to resistance exercise. For endurance exercise, the model predicted increases in JNK and S6 which were not observed experimentally. It predicted decreases in TSC1/2 and MAFbx, which were experimentally observed to increase. Finally, the model failed to predict a significant increase in MuRF1 in response to resistance exercise. These discrepancies will help to identify areas for model refinement in future revisions.

The model results and validation presented here were primarily qualitative. Although the analysis produces continuous results, they are all normalized to between 0 and 1, and we used constant default values for all network parameters, owing to incomplete availability of data for all network nodes and reactions and to avoid over‐parameterization. Computed quantitative changes in signalling nodes, genes and phenotypic outputs are small compared with experimental findings, especially when expressed as a fraction of steady‐state baseline values. In previous studies using this logic‐based ordinary differential equation approach, an arbitrary change in a node value of 0.05 has been used as a threshold for comparison with a statistically significant experimental change (Ryall et al., 2012; Tan et al., 2017). Here, we used a relative change of 5% as the threshold in the model, and a *P*‐value of 0.05 in the experiments taking into account the sign of the change. Ideally, comparisons would be quantitative, since *P*‐values do not account for effect size, but the outputs of this type of model are not suited to comparison with certain experimental measurements such as gene expression that cannot typically be normalized to a maximum value. A new modification of the current modelling method (Cao et al., 2024) does produce model outputs of mRNA expression normalized to baseline. The accuracies we obtained here with independent validation data of 75%–85% are comparable with previous reports using this method in other systems (Ryall et al., 2012; Tan et al., 2017). We also note that, while the validation studies were chosen a priori and not used in the model formulation, their selection was not random or blinded. Since the model is knowledge based and we needed validation data that included measurements of variables in the model itself, the validation papers were often published after many of the formulation papers, and it is unlikely that the authors of the validation studies were unaware of the prior knowledge used to build the network model.

Qualitative and quantitative model accuracy could be improved by adjusting parameters, especially reaction weights and time constants, which we did not attempt to optimize here. Previous uncertainty quantification studies have shown that the reliability of this class of network model is fairly robust to parameter uncertainty (Cao et al., 2020). We investigated the effects of perturbing the two main major adjustable parameters in the model and found that the model accuracy was reasonably robust to parameter uncertainty and that most of the quantitative changes in model results did not affect the qualitative trends. As more exercise signalling measurements become available, confidence in network logic and interactions may be increased. In particular, if we added more gene targets of the transcriptional regulators in the model, the ability to test model outputs more comprehensively and optimize model parameters would both be increased. Similarly, more detailed time course data would allow the model time constants to be optimized. Most of the validation studies used measurements from biopsies taken about 1 h post‐exercise. Since the model is dynamic, it does account for rest time post‐exercise. A model with time constants optimized by making use of time course measurements during and after exercise could also be used to identify optimal timing of future measurements.

The model does not account for the full range of exercise stimuli. It is not clear whether the value and timing of the endurance exercise stimulus alone will be sufficient to discriminate between sprint and endurance training. And the model is not muscle specific and does not distinguish between eccentric and concentric contractions, which can result in differences in protein activation during resistance training (Vissing, Rahbek et al., 2013).

Hence, we need a more detailed understanding of the common and distinct physical and metabolic stimuli differentiating endurance from resistance exercise. A revised version of this model could rely on a combination of more fundamental and muscle‐type specific physical inputs such as muscle perfusion, force and shortening to capture the parameters of exercise with more precision. Finally, muscle exercise responses are the combined result of multiple systems, cell types and biological processes. Most of the measurements reported in the studies used to formulate the model did not include single cell or cell‐type specific measurements. Improved versions of this model could include paracrine signalling between skeletal myocytes and other cell types, metabolic networks, translation of mRNA to protein and feedback to the network itself, and organ‐system interactions.

### 4.3 Conclusions

We constructed and validated a new network model of skeletal muscle cell signalling, which accurately predicts acute responses to resistance and endurance exercise. Sensitivity analysis demonstrated that resistance and endurance training recruit many of the same signalling cascades, in particular the STARS, MAPK, mTOR and calcium pathways. This model synthesizes a wide range of exercise signalling literature and serves as a new tool for understanding signalling interactions and phenotypic adaptations to acute exercise.

Exercise prescription involves many variables including timing, volume, repetitions of endurance and resistance exercise bouts. However, the biological basis of these recommendations and the physiological differences between different training regimens remain poorly understood. This new model of exercise and resistance training responses in skeletal muscle may help to elucidate the differential responses to and interactions between different exercise training prescriptions. Personalized models could potentially be used to identify different combinations and intensities of endurance and resistance training and rest that optimize specific phenotypic responses.

## AUTHOR CONTRIBUTIONS

Annabelle Fowler, Simon Schenk, Samuel R. Ward, Andrew C. Fry, Padmini Rangamani, and Andrew D. McCulloch contributed to the conception or design of this work. Annabelle Fowler, Katherine R Knaus, Stephanie Khuu, Ali Khalilimeybodi, and Andrew D. McCulloch contributed to the acquisition, analysis, or interpretation of data for this work. Annabelle Fowler, Katherine R. Knaus, Stephanie Khuu, Ali Khalilimeybodi, Simon Schenk, Samuel R. Ward, Andrew C. Fry, Padmini Rangamani, and Andrew D. McCulloch contributed to drafting of this work or revising it critically for important intellectual content. All authors have approved the final version of the manuscript and agree to be accountable for all aspects of the work in ensuring that questions related to the accuracy or integrity of any part of the work are appropriately investigated and resolved. All persons designated as authors qualify for authorship, and all those who qualify for authorship are listed.

## CONFLICT OF INTEREST

Simon Schenk is a consultant for Terns Pharmaceuticals Inc. Andrew D. McCulloch is a co‐founder of Insilicomed Inc. and Vektor Medical Inc., which are licensees of UC San Diego intellectual property. There is no relationship between these companies and the research described here.

## FUNDING INFORMATION

We acknowledge support of this work by the Wu Tsai Human Performance Alliance and the Joe and Clara Tsai Foundation.

## Supporting information

Supplementary Table S2. Summary of species, muscle, exercise conditions and measurements for each of the 87 papers used to formulate the model and 9 papers used for validation.

Supplementary Code S3. Jupyter notebook with Python code and parameters.
Supplementary Table S4. Model nodes, reactions, parameters, and literature sources.

Supplement Table S5A–C. Raw data from sensitivity heatmaps in Figure 4a–c.

Supplementary Figure S1. PRISMA diagram of study selection criteria

## Data Availability

The data that support the findings of this study are available from the corresponding author upon reasonable request.
