# Supplementary material for: Network model of skeletal muscle cell signalling predicts differential responses to endurance and resistance exercise training
Source: Exp Physiol. 2024 Apr 21;109(6):939–55. doi: 10.1113/EP091712 (PMC11140181; doi:10.1113/EP091712)
Supplement: Supplementary file 4 — Supplementary Figure S1. PRISMA diagram of study selection criteria [file EPH-109-939-s004.pdf]

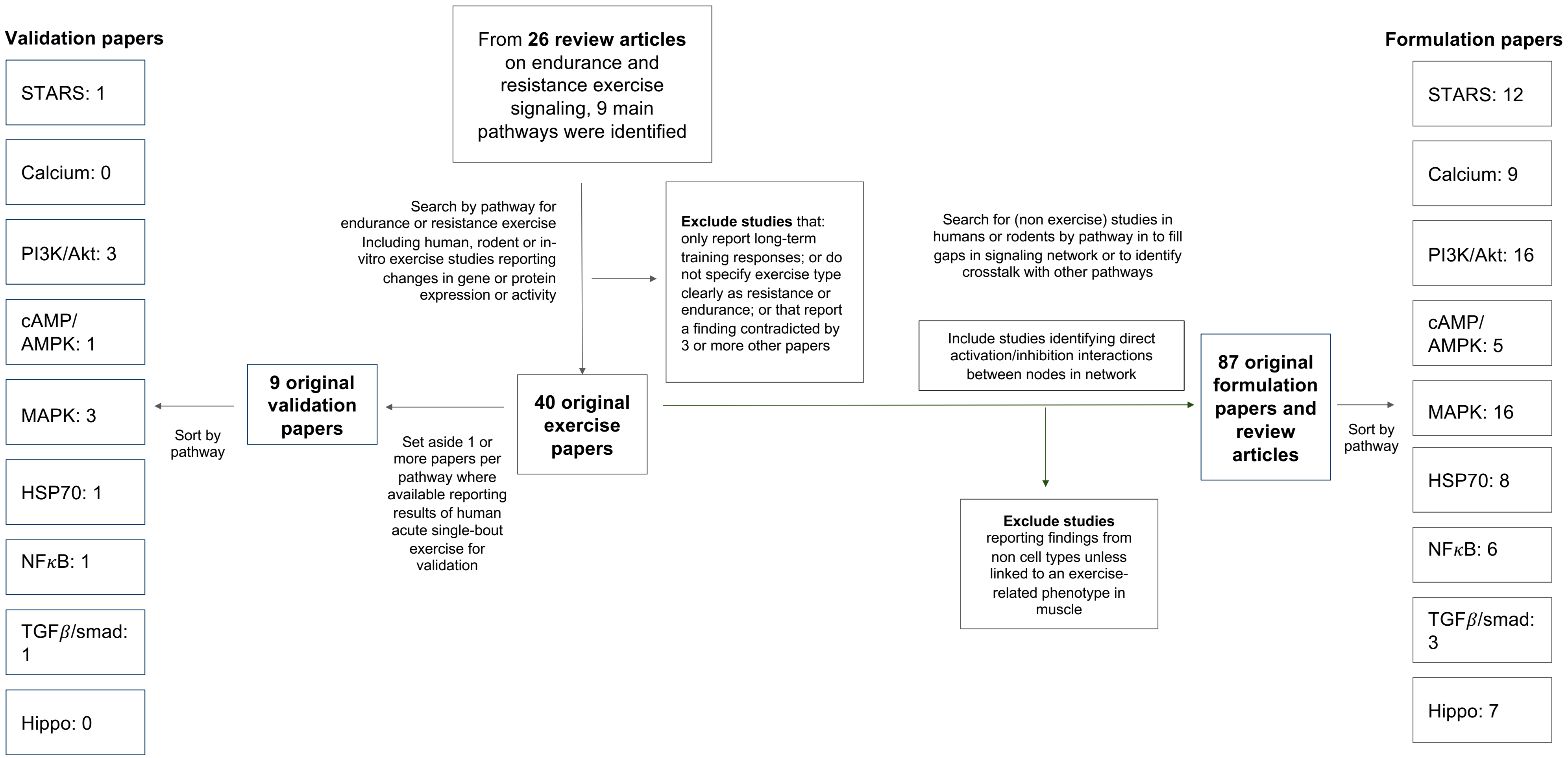
Validation papers
STARS: 1
Calcium: 0
PI3K/Akt: 3
cAMP/ AMPK: 1
MAPK: 3
HSP70: 1
NFκB: 1
TGFβ/smad: 1
Hippo: 0
Sort by pathway
9 original validation papers
From 26 review articles on endurance and resistance exercise signaling, 9 main pathways were identified
Search by pathway for endurance or resistance exercise Including human, rodent or in-vitro exercise studies reporting changes in gene or protein expression or activity
Exclude studies that: only report long-term training responses; or do not specify exercise type clearly as resistance or endurance; or that report a finding contradicted by 3 or more other papers
40 original exercise papers
Set aside 1 or more papers per pathway where available reporting results of human acute single-bout exercise for validation
Search for (non exercise) studies in humans or rodents by pathway in to fill gaps in signaling network or to identify crosstalk with other pathways
Include studies identifying direct activation/inhibition interactions between nodes in network
Exclude studies reporting findings from non cell types unless linked to an exercise-related phenotype in muscle
87 original formulation papers and review articles
Sort by pathway
Formulation papers
STARS: 12
Calcium: 9
PI3K/Akt: 16
cAMP/ AMPK: 5
MAPK: 16
HSP70: 8
NFκB: 6
TGFβ/smad: 3
Hippo: 7
